# Characteristics and Predictors of Abstinence Among Smokers of a Smoking Cessation Clinic in Hunan China

**DOI:** 10.3389/fpubh.2021.615817

**Published:** 2021-03-19

**Authors:** Yina Hu, Jianghua Xie, Xiaochang Chang, Jianhua Chen, Wei Wang, Lemeng Zhang, Rui Zhong, Ouying Chen, Xinhua Yu, Yanhui Zou

**Affiliations:** ^1^Hunan Cancer Hospital/The Affiliated Cancer Hospital of Xiangya School of Medicine, Central South University, Changsha, China; ^2^Hunan University of Chinese Medicine, Changsha, China

**Keywords:** smoking cessation, smoking cessation clinic, quitting predictor, smoker characteristic, smoking, abstinence rates, smoking quit rate, multivariate binary regression

## Abstract

**Background:** More than 300 million smokers make China the largest cigarette consumer globally, which is a huge economic burden. Smoking cessation (SC) clinics can offer counseling and follow-up services. The operational experience of SC clinics in China needs to be summarized and improved based on research evidence.

**Purpose:** The objectives of this study were to describe quit rates among attendees of SC clinics in Hunan and assess predictors of successful SC.

**Methods:** The participants in this study were smokers who visited the SC clinic of Hunan Cancer Hospital from February 1, 2015 to September 30, 2018. Individuals who received individual counseling and assessment from the SC clinic staff and were willing to quit smoking were eligible for inclusion. Those with critical illness or cancer were excluded. Application of smoking cessation clinic registration form (unified by Chinese Center for Disease Control and Prevention) was used to assess participants at the consultation. Follow-ups and counseling were performed over telephone at 1 week, 1 month, and 3 months after the initial cessation consultation or in times of need. Successful SC was checked for at 3 months after the start of SC.

**Results:** A total of 328 smokers (mean age 45.67 ± 12.38 years) had participated. The abstinence rate at 3 months was 28.4%. Binary regression analysis revealed significant independent predictors to be the total numbers of SC follow up sessions, previous SC attempts, and participants' decision on when to quit smoking (The relative to quit immediately group, quit within 30 days, quit after 30 days, and undecided quit were less likely to succeed in quitting. while quit within seven days had no statistical significance.

**Conclusion:** SC clinics can achieve a desirably high quit rate. Participant's previous attempts at quitting, three or more follow-ups, and the decision to quit immediately or within seven days were factors helpful in predicting the success of SC.

## Introduction

Smoking is the leading preventable cause of death worldwide (1). Smoking rates in China remain high; in the past 30 years, some progress in cigarette control has been made in China, with the smoking rate among men dropping from 61% in 1984 to 50.5% in 2018 (2, 3). However, it is still higher than the world average (36.1%) and European average (39.0%) (4). Studies have shown that smoking cigarettes can lead to a variety of malignant tumors, especially lung cancer (5–7). In addition, 10% of cardiovascular deaths can be attributed to smoking (8). Smoking 20 cigarettes daily was found to increase the risk of coronary heart disease by 127% in men and 295% in women. Even one cigarette per day increased the risk of coronary heart disease by 74% in men and 119% in women (9).

Quitting smoking plays a key role in greatly reversing preexisting damage and restoring health (10). People who quit smoking are less likely to have diseases than persistent smokers (11). Studies have shown that smoking cessation (SC) reduces the morbidity and mortality of smokers at any age (12). Smokers who give up smoking before the age of 40 can reduce their risk of mortality from smoking-related diseases by 90% compared to that of persistent smokers (13). For cancer patients, cigarette cessation can improve their prognosis, reduce the risk of a second primary tumor in the future, and increase their lifespan (14). Moreover, SC improves quality of life and longevity while rapidly reducing the risk of heart diseases (15).

Despite extensive smoking control efforts in China, cigarette smoking remains a challenging social and public issue. It has been reported that, without proper guidance and planning, approximately one-third of current Chinese male smokers will die from smoking-related diseases by 2030 (16). More than 40% of smokers attempt to quit every year, and only 3–5% succeed without help from a health care provider (17). However, with cessation aid provided by medical staff, the cessation rate increased (18), and brief advice was shown to increase quitting rates by 1–3% (19). There is evidence that cessation advice and counseling can increase successful quitting rates by ~3% (20). Huang et al. (21) reported that people who quit smoking were assessed over the phone interview and asked to report their quit status.

The WHO recommends offering cessation services as a part of primary health care (22). These services mainly include SC clinics, quit lines, pharmacotherapy, and health professionals who provide brief advice to smokers on quitting smoking as they avail health services (23). SC clinics where physicians and other health care providers give brief, professional advice are necessary to help smokers quit and decrease the health burden of tobacco smoking (24).

Furthermore, it is important for SC professionals to guide cessation based on evidence of predictor success. Predictors of successful SC include factors such as number of previous SC attempts, reasons to quit, setting a SC date, health status, and influence of family members. According to a study in Guangzhou, China, smokers who were confident and took the initiative to quit were more likely to succeed, with a prevalence quit rate of 24% after 6 month and 7 days (25). Jampakla et al. (26) studied 2,000 individuals who quit smoking at the SC clinics in Thailand, showed that age, cigarette consumption, and self-assessed addiction were the factors that reduced relapse. See et al. (27) reported a 27.6% abstinence rate after 6 months among inpatients who quit smoking; Of them, those who had adopted the cold turkey method and faced social pressure were more likely to succeed. Another study revealed that tobacco users with a relatively high socioeconomic status were more likely to have tried to quit (28). Hagimoto et al. (29) reported that the more nicotine dependent a smoker is, the less likely they are to succeed in quitting. A study of women quitting smoking in Saudi Arabia found that the most common reason for quitting was concern for their health, while the least likely reason was the fear that smoking affected their mood (30). Some studies showed that individualized consultation by medical staff improved the SC rates, and that consultation intensity had a strong dose-response relationship with the success rate of smoking cessation; consultation frequency of four or more was the most beneficial (1). A meta-analysis indicated that a brief tobacco intervention was also effective (31). Hence, we can infer that regular follow-up counseling may have a significant positive influence on SC.

SC clinics have been set up in hospitals in major cities in China since 2008. Alongside their effectiveness in improving SC rates, there is also evidence supporting their cost-effectiveness (1). SC clinic operation and experience of clinic attendees need to be summarized to make continuous improvement. In this study, we aimed to describe smoking behavior and determine smoking quit rates among people attending SC clinics. We also aimed to study predictors of successful SC.

## Methods

### Study Setting and Participants

This study was carried out in the SC clinic of Hunan Cancer Hospital (a tertiary cancer teaching hospital), which was set up in 2015. The clinic offers free quit-smoking service on two of the weekday afternoons (Tuesdays and Fridays) and a SC hotline during working hours. Individuals who were willing to quit smoking from February 1, 2015 to September 30, 2018 were included in the study. They came to the clinic by themselves or were referred by doctors. Some participants were also recruited from the communities; clinic nurses visited communities once a month to offer advice to residents about quitting smoking, as well as to recruit participants who wanted to quit smoking. Participants with critical illness such as cancer were excluded. This study was approved by the Ethics Committee of Hunan Cancer Hospital (KYJJ-094). Oral informed consents were obtained from all participants.

### Sample Size Calculation

In this study, the sample size formula required to estimate the population rate is: $n=\frac{u_{\alpha /2}^{2}\pi \left(1-\pi \right)}{\delta^{2}}$. It is known that the smoking cessation rate of patients receiving smoking cessation intervention in smoking cessation clinic of six hospitals in Chengdu is 9.7% at 3 months (32), α = 0.05, U_0.05/2_ = 1.96, δ = 0.05, and the sample size is calculated to be at least 135 cases. Considering the 20% loss of follow-up rate, the estimated sample number should be at least 162 cases. Based on the above literature evidence, the team was only able to select the 3-month smoking cessation rate as the calculation criterion which is relatively low. Before 2013, China's smoking cessation clinics have not been carried out on a large scale. In recent years, the Chinese government has vigorously promoted tobacco control propaganda to improve public health literacy, which is conducive to increasing the smoking cessation rate. Combined with the smoking cessation rate in research of Aung et al. (33), the sample size of this study should be increased predictably.

### Definition and Evaluation of Indicators

Participants were assessed by the SC clinic doctors or nurses and provided with individual counseling at the time of their initial visit or consultation. The first assessment included collecting information on participants' demographic data, nicotine dependence, and willingness to quit smoking. Smoking cessation reasons and motivation are assessed (**Table 2**). The questionnaire used was designed by the Chinese CDC (34).

#### Demographic Information and General Condition

Demographic information of participants included gender, age, occupation, and education level. Participants general condition (well, fair, poor) was assessed by the doctors or nurses of SC clinic.

#### Nicotine Dependence and Smoking Status

Nicotine dependence was assessed according to the Fagerstrom Test for Nicotine Dependence (FTND) (35). Its scores were categorized as the following: 0 as no dependency, 1–3 as low dependency, 4–6 as moderate dependency, and 7–10 as severe dependency. Information about daily cigarette consumption and smoking duration were also collected.

#### CO Level Test

Smokers are given CO tests when they consulted with the SC clinic nurses or doctors. Exhalatory carbon monoxide test value (ppm): 0–6 for non-smoking; 7–10 are light smoking; 11–20 were moderate smoking; 21–72 are heavy smoking (36).

#### Operational Definitions

##### Smokers

According to WHO (35), smokers are defined as those who smoke continuously or accumulatively for 6 months or more in their lifetime. Regular smokers are defined as smokers who smoke ≥ 1 cigarette per day for more than half a year (37).

A former smoker is one who had smoked one cigarette per day for at least 6 months and had quit at the time of the survey (38).

##### Attempts to Quit Smoking

It includes any attempt by a smoker to quit smoking within the past 12 months (39).

##### Assessment of Smoking Cessation

After the initial consultation, standardized telephone follow-up assessments were conducted by the same group of trained investigators at 1 week, 1 month, and 3 months. The abstinence time was calculated as beginning from the day the individual stopped smoking to the day of the assessment. Smokers who did not go to the SC clinic in person were assessed by telephonic interview (21). Rather than directly asking whether they had quit smoking, they were asked how many cigarettes they smoked per day. Those who answered that they no longer smoked were considered successful quitters.

##### Success in Smoking Cessation

This was decided based on the self-report of smokers who have maintained their smoking cessation status from the beginning of treatment until the end (34).

### Assistance in Quitting

#### Face-to-Face Consultation

Trained nurses and doctors provided face-to-face SC counseling for 20–30 min during the first consultation of the participants.

#### Behavioral Support

Detailed counseling and a brief advice (19, 40) was given to participants during consulting and telephonic interview, respectively. The 5As/5Rs, given by Prochaska and Goldstein (41), were also applied to SC. The “5As” refers to ask, advice, appraise, assistance, and arrangement. The “5Rs” are measures that enhance the motivation to quit smoking; they include relevancy, risk, reward, road block, and repeat.

#### Follow Up Guidelines

At least three follow-up sessions took place over the telephone, at 1 week, 1 month, and 3 months, after the initial visit by clinic nurses to the community. Follow-ups were provided according to the individual's needs. With every follow up, behavioral support and health education were given to the participant. Additionally, the staff also filled out the standard evaluation form at the three follow-ups. According to the results of these three assessments, the help required by the participants was determined: (1) for those who had successfully adhered to the goal of SC, medical staff provided guidance for successfully dealing with withdrawal symptoms and encouraged participants to continue cessation in order to increase their confidence and prevent relapse; (2) for smokers who had not yet quit, medical staff tried to understand the resistance to SC and emphasized the harm of smoking and benefits of SC; and (3) participants who relapsed after quitting were encouraged to use medication or given advice about another attempt.

#### Lost to Follow-Up

Those whose telephone numbers were wrong and who never answered were counted as lost participants at the first follow up. Those whose telephone numbers could be rung but who did not answer after seven or more times were considered to have failed in SC.

#### Medication Therapy

SC medication therapy is advised for people who want to quit smoking (23). SC doctors prescribed the medication for those who were willing to quit if the participants agreed.

### Statistical Analysis

Data were entered and analyzed using SPSS version 18.0 (SPSS, Chicago, IL, USA). Descriptive statistics were calculated for demographic information and smoking status. We performed univariate analyses with chi-square tests between the SC success group and the SC failed group. A binary regression analysis was used to identify the factors predicting 3 months of cessation. Odds ratios (ORs) and 95% confidence intervals (CIs) were evaluated in multivariable analysis. A *P*-value < 0.05 denoted statistical significance.

## Results

### Overall SC Rate and the Participant Baseline Characteristics

**Figure 1:**
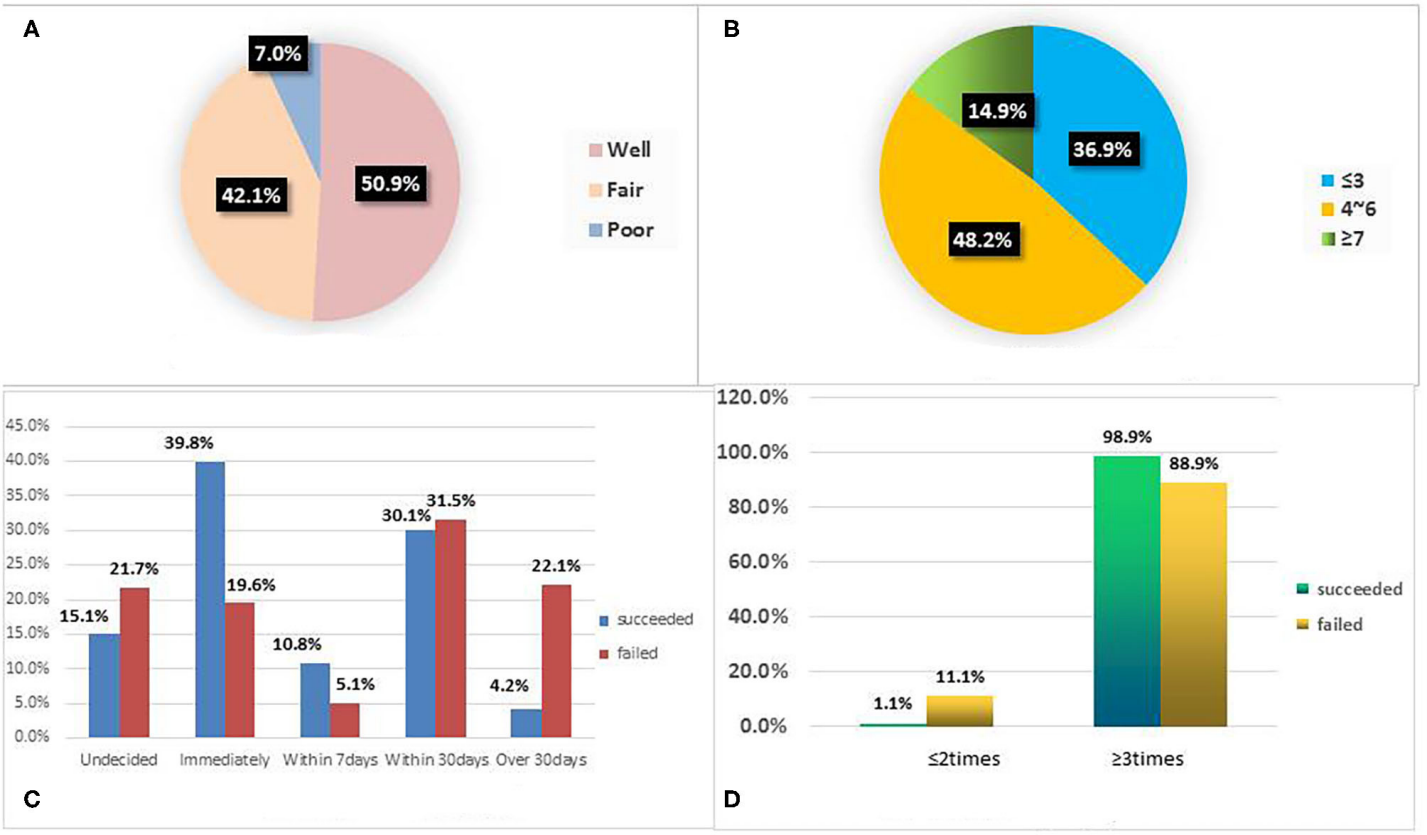
**(A)** Health assessment represents the proportion of the subjective assessment of physical condition grade of 328 patients; **(B)** Fagerstrom score represents the proportion of Fagerstrom Test for Nicotine dependence among 328 patients. **(C)** Date of SC represents the proportional distribution characteristics of the expected start time of smoking cessation between the successful and unsuccessful smoking cessation groups; **(D)** Follow ups represents the proportional distribution characteristics of the number of follow-up visits between the successful group and the failed group. SC, smoking cessation.

A total of 335 participants were enrolled in this study. Seven participants with incomplete data were excluded, and the final sample included 328 participants. We were unable to contact 12 participants among them; the lost to follow-up rate was 3.67% (12/328). The overall SC rate at 3 months was 28.4% (93/328). There were 97.6% male participants (320/328) and only 2.4% (8/328) female participants. The mean age of the participants was 45.67 ± 12.38 years. The youngest and oldest participants were 18 and 80 years old, respectively. In terms of education, 8.2, 31.7, 27.2, and 32.9% of the participants had primary school or less, junior high school, senior high school, and college education or above, respectively. The most common occupation was enterprise/business/service personnel (39.6%), followed by farmers (24.1%). In terms of health assessment, 50.9% (167/328) participants' general condition was well, while 7.0% (23/328) were in poor condition (Figure 1A). Univariate analysis showed that there was no significant difference in gender, age, education level, occupation, duration of smoking habit, daily cigarette consumption, nicotine dependence score, CO test, and SC aids between the SC success group and the SC failed group (*P* > 0.05). However, the total number of SC follow up sessions (*P* = 0.003), the number of previous SC attempts (*P* = 0.002), health assessment (*P* = 0.031), and date of SC (*P* < 0.001) were significantly different between the two groups (Table 1).

**Table 1:** Clinical characteristic of participants between success group and failure group in SC for 3 months.

**Variables**	**Total (*n* = 328)**	**Abstinence succeeded (*n* = 93)**	**Abstinence failed (*n* = 235)**	**χ^**2**^**	***P***
Gender				0.034^a^	0.854
Male	320 (97.6)	90 (28.1)	230 (71.9)		
Female	8 (2.4)	3 (37.5)	5 (62.5)		
Age (y)	A:45.67 ± 12.38			1.797	0.407
18–40	110 (33.6)	32 (29.1)	78 (70.9)		
41–59	167 (50.9)	43 (25.7)	124 (74.3)		
≥60	51 (15.5)	18 (35.3)	33 (64.7)		
Education Level				1.943	0.584
Primary school or less	27 (8.2)	5 (18.5)	22 (81.5)		
Middle school	104 (31.7)	28 (26.9)	76 (73.1)		
High school	89 (27.2)	26 (29.2)	63 (70.8)		
College school or above	108 (32.9)	34 (31.5)	74 (68.5)		
Occupation				5.660	0.462
Teachers	7 (2.1)	2 (28.6)	5 (71.4)		
Medical staff	21 (6.4)	8 (38.1)	13 (61.9)		
Government/institution staff	27 (8.2)	9 (33.3)	18 (66.7)		
Retired/unemployed	31 (9.5)	13 (41.9)	18 (58.1)		
The others	33 (10.1)	9 (27.3)	24 (72.7)		
Farmers	79 (24.1)	18 (22.8)	61 (77.2)		
Enterprise/business/service personnel	130 (39.6)	34 (26.2)	96 (73.8)		
Health Assessment				6.936	0.031
Well	167 (50.9)	45 (26.9)	122 (73.1)		
Fair	138 (42.1)	36 (26.1)	102 (73.9)		
Poor	23 (7.0)	12 (52.2)	11 (47.8)		
Duration of smoking (y)	A: 22.81 ± 12.32			1.377	0.711
≤10	73 (22.3)	23 (31.5)	50 (68.5)		
11–20	94 (28.7)	24 (25.5)	70 (74.5)		
21–30	90 (27.4)	28 (31.1)	62 (68.9)		
≥31	71 (21.6)	18 (25.4)	53 (74.6)		
Daily cigarettes consumption	A: 21.85 ± 11.36			2.587	0.460
≤10	61 (18.6)	22 (36.1)	39 (63.9)		
11–20	175 (53.4)	48 (27.4)	127 (72.6)		
21–30	52 (15.9)	14 (26.9)	38 (73.1)		
≥31	40 (12.1)	9 (22.5)	31 (77.5)		
SC attempts previously				9.282	0.002
Yes	212 (64.6)	72 (34.0)	140 (66.0)		
No	116 (35.4)	21 (18.1)	95 (81.9)		
Fagerstrom score	A: 4.27 ± 2.12			2.128	0.345
≤3	121 (36.9)	40 (33.1)	81 (66.9)		
4–6	158 (48.2)	41 (25.9)	117 (74.1)		
≥7	49 (14.9)	12 (24.5)	37 (75.5)		
CO test results				0.881	0.644
0–5	37 (22.1)	8 (21.6)	29 (78.4)		
6–10	25 (15.0)	8 (32.0)	17 (68.0)		
11–72	105 (62.9)	29 (27.6)	76 (72.4)		
Date of SC				20.512	0.000
Undecided	65 (19.8)	14 (21.5)	51 (78.5)		
Immediately	83 (25.3)	37 (44.6)	46 (55.4)		
Within 7 days	22 (6.7)	10 (45.5)	12 (54.5)		
Within 30 days	102 (31.1)	28 (27.5)	74 (72.5)		
Over 30 days	56 (17.1)	4 (7.1)	52 (92.9)		
Follow up	A:2.81 ± 0.66			8.800	0.003
≤2	27 (8.2)	1 (3.7)	26 (96.3)		
≥3	301 (91.8)	92 (30.6)	209 (69.4)		
SC adds					0.131^*^
BS	316 (96.4)	87 (27.5)	229 (72.5)		
BS and Medication	6 (1.8)	4 (66.7)	2 (33.3)		
BS and EC	6 (1.8)	2 (33.3)	4 (66.7)		

*A, mean ± standard deviation*,

a *continuous correction Value; SC, smoking cessation; BS, behavior support; EC, electronic cigarettes*.

* *Monte Carlo P*.

### Participants' Smoking Behavior Prior to Consultation

Among the current smokers, the average duration of smoking was 22.81 ± 12.32 years, and the average consumption per day was 21.85 ± 11.36 cigarettes. A significant proportion of smokers (77.7%) had smoked for more than 10 years, with the longest smoking period being 60 years. Over 80% participants smoked 10 cigarettes or more per day, with the highest proportion of participants smoking 11–20 cigarettes per day (53.4%).

A total of 64.6% participants (212/328) had previously made quitting attempts. The SC success group had a higher rate of previous attempts than the SC failed group (77.4 and 59.6%, respectively) (Table 1).

### Nicotine Dependence Score and CO Tests Results

FTND assessment results showed an average nicotine dependence score of 4.27 ± 2.12 (Figure 1B). CO tests were performed using a gas detector on 167 participants who were willing to accept the procedure. Results showed that heavy smokers and light smokers accounted for 80.7% (105/130) and 19.3% (25/130) of the participants who received the CO test, respectively (Table 1).

### The Quitting Date and the Methods Used to Assist in Quitting Smoking

In relation to the quitting dates of the 328 participants, 25.3% (83/328) quit immediately, 6.7% (22/328) quit within seven days, 31.1% (102/328) quit within 30 days, 17.1% (56/328) decided to quit after 30 days, and 19.8% (65/328) did not decide when to quit (Figure 1C).

Every participant received at least one counseling session. In the SC success group, 98.9% (92/93) participants received three or more follow-ups. For the SC failure group, this figure was 88.9% (209/235). The mean follow-up frequency was 2.81 ± 0.66 (Figure 1D). During the 3-month SC follow-up, 96.4% (316/328) of the participants received only behavioral support interventions, 1.8% (6/328) received pharmacotherapy and behavioral support interventions, and 1.8% (6/328) chose e-cigarette as a means of self-assisted SC in addition to behavioral support interventions (Table 1).

### Reasons and Motivations for Quitting Smoking

The top four reasons and motivations for quitting smoking were the following: (1) awareness of the hazards of smoking and wanting to return to a healthy life, (2) belief that smoking was causing a gradual decline in one's health, (3) family demands, and (4) knowing someone who was sick from smoking. They accounted for 74.4% (224/328), 24.4% (80/328), 19.5% (64/328), and 15.9% (52/328), respectively. Other reasons and motivations to quit smoking were that their friends disliked them for smoking, they could afford to quit, and that they could not smoke in places that were non-smoking for fear of causing trouble among other reasons. There was no statistically significant difference in the analysis of smoking cessation reasons and motivation between the SC successful group and the SC failure group (*P* > 0.05) (Table 2).

**Table 2:** Univariate analysis of smoking cessation reasons and motivation of participants.

**Variables**	**Total (*n* = 328)**	**Succeeded (*n* =93)**	**Failed (*n* = 235)**	**χ^**2**^**	***P***
Knowing the hazards of smoking					
No	84 (25.6)	29 (34.5)	55 (65.5)	2.116	0.146
Yes	224 (74.4)	64 (26.2)	180 (73.8)		
Decline in health				2.301	0.129
No	248 (75.6)	65 (26.2)	183 (73.8)		
Yes	80 (24.4)	28 (35.0)	52 (65.0)		
Knowing somebody sick because of smoking				0.847	0.357
No	276 (84.1)	81 (29.3)	195 (70.7)		
Yes	52 (15.9)	12 (23.1)	40 (76.9)		
Family asks to quit smoking				3.274	0.070
No	264 (80.5)	69 (26.1)	195 (73.9)		
Yes	64 (19.5)	24 (37.5)	40 (62.5)		
People around me hate me smoking				0.007	0.931
No	290 (88.4)	82 (28.3)	208 (71.7)		
Yes	38 (11.6)	11 (28.9)	27 (71.1)		
Have ability to quit smoking myself				0.000	1^a^
No	313 (95.4)	89 (28.4)	224 (71.6)		
Yes	15 (4.6)	4 (26.7)	11 (73.3)		
Avoid the hassles caused in non-smoking places				0.000	1^a^
No	316 (96.3)	90 (28.5)	226 (71.5)		
Yes	12 (3.7)	3 (25.0)	9 (75.0)		
Take part in sports					0.195^⋆^
No	325 (99.1)	91 (28.0)	234 (72.0)		
Yes	3 (0.9)	2 (66.7)	1 (33.3)		
Avoid conflicts with family				0.021	0.885^a^
No	313 (95.4)	88 (28.1)	225 (71.9)		
Yes	15 (4.6)	5 (33.3)	10 (66.7)		
Avoid trouble at school/workplace				0.000	1^a^
No	320 (97.6)	91 (28.4)	229 (71.6)		
Yes	8 (2.4)	2 (25.0)	6 (75.0)		
Improve grooming and eliminate bad odors				0.513	0.474^a^
No	316 (96.3)	88 (27.8)	228 (72.2)		
Yes	12 (3.7)	5 (41.7)	7 (58.3)		
Increase appetite/weight				0.000	1^a^
No	321 (97.9)	91 (28.3)	230 (71.7)		
Yes	7 (2.1)	2 (28.6)	5 (71.4)		
The Others				0.533	0.465^a^
No	322 (98.2)	90 (28.0)	232 (72.0)		
Yes	6 (1.8)	3 (50.0)	3 (50.0)		
Number of reasons and motivations for quitting					0.833^*^
0	16 (4.9)	3 (18.8)	13 (81.2)		
1	205 (62.5)	60 (29.3)	145 (70.7)		
2	51 (15.5)	15 (29.4)	36 (70.6)		
≥3	56 (17.1)	15 (26.8)	41 (73.2)		

a *Continuity correction*,

* *The monte carlo P-Value*,

⋆ *Fisher P-Value*.

### The Predictor Factors of SC

The factors with statistical significance in univariate analysis were included in binary logistic regression to assess the predictors of successful SC at 3 months. We found that previous SC attempts, date of SC, and follow-ups were independent predictors of a 3-month success in SC. The results showed that smokers who had three or more follow-ups were more likely to quit smoking than those who had no more than two follow-ups (OR = 13.266, 95% CI: 1.725–101.991, *P* = 0.013). Compared with those who had never tried to quit before, participants who had made previous attempts at quitting were more than twice as likely to quit (OR = 2.070, 95% CI: 1.155–3.709, *P* = 0.015). Those who chose to quit smoking within 30 days (OR = 0.430, 95%CI: 0.229–0.810, *P* = 0.009), after 30 days (OR = 0.103, 95%CI: 0.034–0.317, *P* < 0.001), and those who did not decide when to quit smoking (OR = 0.370, 95%CI: 0.173–0.788, *P* = 0.010) were less likely to quit smoking than those who quit immediately (Table 3).

**Table 3:** Smoking cessation predictive factors multivariate binary logistic regression analysis.

**Variable**	**β**	**SE**	**Waldχ^2^**	***P***	**Estimated odds ratio**	**95% confidence interval**
**Previous SC attempts**						
No				1		
Yes	0.727	0.298	5.975	0.015	2.070	1.155–3.709
**Date of SC**						
Date of SC (Immediately)				1		
Date of SC (Within 7 days)	0.028	0.503	0.003	0.956	1.028	0.384–2.757
Date of SC (Within 30 days)	−0.843	0.323	6.823	0.009	0.430	0.229–0.810
Date of SC (After 30 days)	−2.270	0.572	15.747	0.000	0.103	0.034–0.317
Date of SC (Undecided)	−0.995	0.386	6.647	0.010	0.370	0.173–0.788
**Follow ups**						
≤2				1		
≥3	2.585	1.041	6.171	0.013	13.266	1.725–101.991

## Discussion

### Characteristics of SC Participants

Most participants of this study were male (97.6%), which is similar to the participant characteristics of other studies (42–44). Gender differences may be related to the low smoking rate among women in Chinese (2.1%) (2). Similar low smoking rates among women have been observed in other countries. Vietnam's demographic surveillance system showed that men and women have a smoking rate of 34.7 and 0.9%, respectively (45). In a cross-sectional study in rural Andhra Pradesh, India, the smoking rate among men was significantly higher (50.3%) than among women (4.8%), but the SC rate among women (45.5%) was significantly higher than that among men (18.8%) (46). Giovino et al. 's (42) analysis of data from 14 low and middle-income countries showed that men in Global Adult Tobacco Survey (GATS) countries have a smoking rate of 48.6% and women 11.3%.

In this study, univariate analysis showed no statistically significant difference between age bracket and success rate of SC. Some studies have suggested that age is a factor in the success of quitting, while others have found the opposite (21, 47–49). Further research is needed to determine the role of age as a factor in SC success.

The attendance rate of smokers at our SC clinics was lower than that in the study by Hamadeh et al. (50) in Bahrain, which was attended by more than 100 patients monthly. Patients' attendance in consulting a health professional is dependent on their willingness to quit smoking as well as their access to SC resources. One of the main reasons for the low attendance rate of smokers in this study can be that Hunan is a large province, and it is not convenient for participants from all over the province to come to the hospital. To strengthen interventions for SC, future studies are advised to use mobile phones or other electronic devices, which are convenient for interaction and reinforcement for both the professional and the smokers (51, 52).

### The Relationship Between Reasons, Motivation, and Success in Quitting Smoking

In this study, the main reasons and motivations for the patients to quit smoking included recognizing smoking as a health hazard, knowing someone sick from smoking, family demands, and believing that smoking led to a gradual decline in their health status. The following literature also proves the result of this study, Wang et al. (53) suggested that the direct or indirect health issues experienced by smokers were the main motivation for quitting smoking, which was also an important reason for smokers to succeed in quitting smoking. A cohort study of adult smokers in six European Union countries showed that focus on individual health and paying close attention to related diseases caused by smoking were important reasons for quitting smoking. Factors such as affecting the health of non-smokers, health care professionals' advice, and the opposition of friends and family were considered important reasons to stop smoking, but were not successful predictors of SC (54). Thomas et al. (55) believed that health problems were one of the most common reasons for smokers to quit, and while the reasons to quit could not predict ultimate success in SC, it led the smoker to continue to try to quit. A study in Singapore (27) showed that smokers who cited social pressures from by family or friends as quitting motivation were more likely to quit (*P* < 0.001), and quitting due to social pressure as quitting motivation (OR = 1.35, 95%CI = 1.10–1.66, *P* = 0.005) remained an independent predictor of successful quitting. However, Klemperer et al. (56) conducted an intervention on motivation of people who were initially reluctant to quit smoking and found that the increase of motivation to quit could predict the smokers' attempts to quit smoking and their success in quitting. Pineiro et al. (57) found that smokers' motivation to quit can predict short- and long-term success in quitting, as well as the maintenance of this long-term success, which is consistent with reports from other studies (58, 59).

### Success Rate of SC

Our study showed that the participants' 3-month SC rate was 28.4% (93/328). This is higher than usual; success rate of SC is generally <10% (17). This could be because SC rates vary depending on the method used. The SC clinic in this study offered both face-to-face counseling and follow-up through telephone. In China, the quit rate in the general population without any aids is <5% (2). The SC intervention in primary health care units in Thailand had a continuous smoking cessation rate of 25.62% at 6 months (33). A research in Mumbai, India, reported that the women's community SC program used health education, games, and follow-up counseling sessions to intervene once every 3 months, and the SC rate at 12 months was 33.5% (60). Another study conducted in India reported a rate of merely 2.6% (61). SC clinics may encourage tobacco control efforts and help decrease the health burden of smoking.

### Predictors of SC

Binary logistic regression predictor analysis revealed that the starting date of SC, the number of previous quitting attempts, and the number of follow-up after the first consultation were independent predictors of smoking cessation success (*P* < 0.05). However, the duration of smoking, daily cigarettes consumption, Fagerstrom score, and exhaled CO value at first visit were not associated with successful smoking cessation in this study. Ma et al. (62) showed that lower nicotine dependence level, and counseling sessions received were significantly associated with success in quitting smoking (*P* < 0.01). Shie et al. (63) found that smokers with the higher intention to quit was more likely to quit smoking successfully (*P* < 0.001). Huang et al. analyzed smoking cessation rate in a hospital smoking cessation clinic in Taiwan, showed that the independent predictors of smoking cessation success were the average number of cigarettes smoked each day, nicotine dependence level, exhaled CO value at first visit, and number of clinic visits (*P* < 0.05) (21). However, a study on smoking cessation clinic of a hospital in inland China (Ningbo City) showed that the number of outpatient visits was the most important factor for success in smoking cessation, while the CO level exhaled at the first visit was not associated with success in smoking cessation (64). These results all confirm that the number of counseling sessions after starting smoking cessation is an independent predictor of success, with some variation in other predictors. These different findings arise and require further exploration.

#### Previous Attempts to Quit Smoking Enhance Quit Rate

Approximately 64.6% of current smokers in this study had attempted to quit smoking previously. The result also indicated that previous attempts to quit smoking were positive factors for SC success and a greater number of attempts to quit smoking increased the possibility of SC success, which is consistent with another study in China (65). Other studies reported that SC intentions and SC attempts were also important indicators for predicting future SC attempts and success rates (48, 66). A study of five smoking cessation clinics in Malaysia suggested that smokers who have tried to quit smoking for longer are more likely to quit (67). At the same time, related studies have shown that the duration of smoking cessation attempt is related to the success of quitting smoking (68, 69). A study exploring patterns and predictors of cessation success among adult smokers in Thailand showed that smokers who tried to quit for more than a week predicted short-term success in quitting (26). In a study of smokers' quitting behavior in six Chinese cities, a history of quitting for longer than 6 months was also an independent predictor of smoking cessation (69). A Bangladeshis study showed that both smokers who had tried to quit 6 months earlier (OR = 0.23, 95% CI:0.09–0.62) and those who had never tried to quit (OR = 0.75, 95% CI:0.34–1.66) were less likely to quit than those who had tried within the last 6 months (70). These results imply that the SC strategies are therefore needed to motivate and encourage smokers to become more ready to quit.

#### Behavioral Support and Follow-Up Are Important Factors for Successful SC

All participants in this study received 20–30 min face-to-face detailed counseling and 0–3 telephone follow-up sessions. Additionally, 1.8% of the participants received behavioral support interventions plus pharmacotherapy and 1.8% were using e-cigarettes alongside behavioral support interventions during SC procedure. Pharmacotherapy can increase the success rate of quitting smoking (23, 40). It is considered an important factor affecting SC success in another study (71), with the withdrawal rate reaching 44.0% at 9–12 weeks (72, 73). However, in our study, it was not a factor affecting SC. We found that the proportion of participants using SC products was 1.8%. Hence, the effectiveness of pharmacotherapy may be underestimated in our study.

A reason for fewer participants choosing pharmacotherapy as preferred intervention was likely because Chinese people are less interested in SC drugs (74). Furthermore, these medications are not covered by medical insurance in China and are often too expensive for smokers to afford. Although there is evidence that e-cigarettes may help smokers quit (75), the WHO has clearly stated that e-cigarettes are harmful, and they may lead to nicotine addiction in teenagers (76). The proportion of successful quitters receiving three or more follow-up sessions was significantly higher than that of unsuccessful quitters [98.9% (92/93) vs. 88.9% (206/235)], which is consistent with the findings of previous studies (77, 78). Two studies in Malaysia suggest that the clinic follow-up sessions was a strong predictor of successful cessation, attending more than one follow-up session increased the chance of success (79, 80). Rabius et al. (81) also found that five psychological interventions together with two telephone follow-up interventions had a 4.1% higher SC rate than only five psychological interventions. A rehabilitation center in Munich, Germany, reported that the SC rate reached with the first psychological intervention alone was 16.8%. With the addition of a follow-up SC intervention, the rate was 30.3% (82). Therefore, follow-up to help smokers quit smoking is very important.

#### Relation Between Setting the Quitting Date and SC Success

The time taken to quit smoking was also associated with success in quitting (*P* < 0.05); the shorter the time was set, the higher the likelihood of SC success. Compared to those who quit immediately, those who were undecided or decided to quit within 30 days or later have a lower success rate of quitting. Li et al. (69) investigated the quitting behavior of adult smokers in six Chinese cities, and the results showed that those who planned to quit smoking within 1 month were more than twice as likely to succeed as those who did not (OR = 2.46, 95% CI: 1.61–3.75), similar to the results of research in Southeast Asia (Malaysia and Thailand) (OR = 2.18, 95% CI: 1.46–3.27) (68). Quit-date goal setting is an important element of an effective brief SC intervention. The quit-date goal setting enhances quit attempts (83). Complete abstinence in the first week after the quit date was a strong predictor of long-term success (84). Health professionals should promptly alleviate the concerns of smokers and help them develop a quality quit-date and an immediate quit plan.

## Limitations

Only the short-term abstinence rate (12 weeks) was reported in our study. The long-term abstinence rate (13–52 weeks) needs to be further evaluated. Moreover, SC success was evaluated by the researcher only according to participants' report that was obtained through telephonic interview; this might lead to biased results.

## Conclusion

In conclusion, in this study, a SC clinic affected SC rate in clinical practice. Smokers receiving regular follow-up counseling for 3 months at the SC clinic exhibited an improved SC rate (28.4%) compared with that of other smokers. Participants who received regular SC counseling were more likely to quit smoking successfully than those who did not receive further counseling. Smokers who had previously tried to quit and who were in a poor physical condition were also more likely to quit. It is suggested that motivation enhancement, cognitive behavioral strategies, and persistent counseling be combined to improve the quit rate. Moreover, the results indicate the need to develop and strengthen SC outpatient services, not only in Hunan Province but also in other parts of China and other countries to obtain a significant decrease in smoking rates.

## Data Availability Statement

The original contributions presented in the study are included in the article/supplementary material, further inquiries can be directed to the corresponding author/s.

## Ethics Statement

The studies involving human participants were reviewed and approved by the Ethics Committee of Hunan Cancer Hospital. Written informed consent for participation was not required for this study in accordance with the national legislation and the institutional requirements.

## Author Contributions

YNH, JHX, and RZ wrote this article. XCC, JHC, WW, and YHZ supervised the whole study and critically revised the paper. LMZ, RZ, XHY, and OYC read and amended the final manuscript. All authors contributed to the article and approved the submitted version.

## Conflict of Interest

The authors declare that the research was conducted in the absence of any commercial or financial relationships that could be construed as a potential conflict of interest.
